# “A FRAIL LITTLE THING, BUT SHE’S WILEY”: POLICE BRUTALITY MEETS DEMENTIA: A NARRATIVE INQUIRY

**DOI:** 10.1093/geroni/igad104.2905

**Published:** 2023-12-21

**Authors:** Kelly Marnfeldt

**Affiliations:** University of Southern California, Los Angeles, California, United States

## Abstract

From 2000 to 2020 overall arrests in the U.S. declined, but arrests of people 65 and older increased by nearly 30 percent. In addition, between 2010 and 2020 approximately 12,000 adults 65 and older were admitted to the emergency room due to injuries sustained during encounters with police or private security. To illuminate the scope of negative consequences that may occur during such encounters, the present study examined the 2020 arrest of a 73-year-old community-dwelling woman living with dementia who walked out of a big-box store with $13.88 worth of merchandise she did not pay for. During her arrest, she sustained multiple injuries at the hands of two officers, including a dislocated shoulder and a broken arm. Despite her injuries, she did not receive medical care for six hours while in custody. The methodological approach to this case draws on elements of interpretative phenomenological analysis, and narrative analysis to explore publicly available archival data sources, including surveillance and police body-worn camera footage. Supporting data sources include police filings at the time of her arrest and legal filings by a civil rights attorney representing the woman’s family. The analysis demonstrated that deeply entrenched ageism, failure to recognize the signs of dementia, and the systemic normalization and defense of police use of force against non-normative behavior when interacting with neuro diverse older adults. This has critical implications for family caregivers and trusted others who struggle to balance the dialectic between safety and independence for people aging in place with dementia

